# Metabolomic analysis of percutaneous fine-needle aspiration specimens of thyroid nodules: Potential application for the preoperative diagnosis of thyroid cancer

**DOI:** 10.1038/srep30075

**Published:** 2016-07-21

**Authors:** Inseon Ryoo, Hyuknam Kwon, Soo Chin Kim, Seung Chai Jung, Jeong A Yeom, Hwa Seon Shin, Hye Rim Cho, Tae Jin Yun, Seung Hong Choi, Chul-Ho Sohn, Sunghyouk Park, Ji-hoon Kim

**Affiliations:** 1 Department of Radiology, Korea University Guro Hospital, Seoul, Korea; 2 College of Pharmacy, Natural Product Research Institute, Seoul National University, Seoul, Korea; 3 Department of Radiology, Seoul National University Healthcare system Gangnam center, Seoul, Korea; 4 Department of Radiology and Research Institute of Radiology, University of Ulsan college of Medicine, Asan Medical Center, Seoul, Korea; 5 Department of Radiology, Pusan National University Yangsan Hospital, Yangsan, Korea; 6 Departement of Radiology, Gyeongsang National University Hospital, Gyeongsang National University School of Medicine, Jinju, Korea; 7 Department of Radiology, Seoul National University Hospital, College of Medicine, Seoul, Korea; 8 Center for Nanoparticle Research, Institute for Basic Science (IBS), Seoul, Korea

## Abstract

Thyroid nodules are a very common problem. Since malignant thyroid nodules should be treated surgically, preoperative diagnosis of thyroid cancer is very crucial. Cytopathologic analysis of percutaneous fine-needle aspiration (FNA) specimens is the current gold standard for diagnosing thyroid nodules. However, this method has led to high rates of inconclusive results. Metabolomics has emerged as a useful tool in medical fields and shown great potential in diagnosing various cancers. Here, we evaluated the potential of nuclear magnetic resonance (NMR) analysis of percutaneous FNA specimens for preoperative diagnosis of thyroid cancer. We analyzed metabolome of FNA samples of papillary thyroid carcinoma (n = 35) and benign follicular nodule (n = 69) using a proton NMR spectrometer. The metabolomic profiles showed a considerable discrimination between benign and malignant nodules. Receiver operating characteristic (ROC) curve analysis indicated that seven metabolites could serve as discriminators (area under ROC curve value, 0.64–0.85). These findings demonstrated that NMR analysis of percutaneous FNA specimens of thyroid nodules can be potentially useful in the accurate and rapid preoperative diagnosis of thyroid cancer.

Thyroid nodules are a very common problem, and they have been detected in up to 67% of the general population with ultrasonography (US) evaluations^1^,^2^,^3^. Cytopathologic analysis of percutaneous fine-needle aspiration (FNA) specimens is the current gold standard for diagnosing thyroid nodules^4^.

However, this procedure has led to high rates of inconclusive results, including nondiagnostic and atypia of undetermined significance or follicular lesion of undetermined significance^5^,^6^,^7^. For these inconclusive thyroid nodules, the Bethesda system and the majority of guidelines recommend repeat FNA^4^,^5^,^8^. Nevertheless, up to 50% of these repeated examinations continue to yield insufficient or indeterminate results^6^,^7^,^9^. In addition, the false negative rate of cytopathologic analysis has been reported to be up to 10.2%^10^.

Due to the limitations of conventional cytopathologic analysis, several molecular analyses have been assessed and reported to have some potential as adjuncts to conventional cytopathologic analysis^11^,^12^,^13^,^14^. However, the results are still unsatisfactory, especially in terms of low sensitivity, negative predictive value, and high cost.

Metabolomics has emerged as a useful tool in multiple medical fields. It has shown great potential in diagnosing various cancers, such as breast, prostate, and colorectal cancers^15^,^16^,^17^,^18^,^19^,^20^. Several articles have presented the potential usefulness of the metabolomic approach for diagnosis of thyroid nodules^21^,^22^,^23^,^24^,^25^,^26^,^27^. Other than *in vivo* proton magnetic resonance spectroscopy (^1^H MRS), which might have limited clinical use, all of the previous studies were based on postoperative surgical specimens. The previous studies did not test the metabolomic approach as a preoperative diagnostic tool for thyroid nodules^21^,^22^,^23^,^24^,^25^,^26^,^27^. Post-operative specimens may not reflect the actual metabolic signature of thyroid nodules analyzed by the current gold standard, FNA before operation.

Therefore, the purpose of this study was to evaluate the potential of nuclear magnetic resonance (NMR) analysis of percutaneous FNA specimens for preoperative diagnosis of thyroid cancer.

## Results

### Cytopathologic results of percutaneous FNA samples

**Figure 1:**
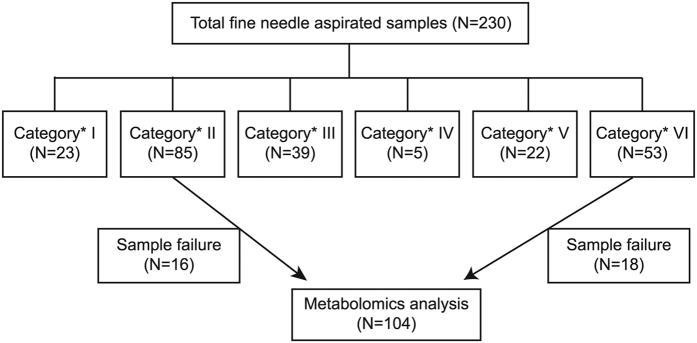
Flow chart of the study group. ^*^Categories were defined according to Bethesda classification.

Among a total of 230 percutaneous FNA samples, 85 were benign (category II; all consistent with benign follicular nodules) and 53 were malignant (category VI; all papillary thyroid carcinomas) based on cytopathologic analyses according to the Bethesda system^5^ (Fig. 1).

Among them, 34 could not be analyzed due to the following reasons:

Thirty-four thyroid nodules (16 benign nodules and 18 malignant nodules) were not analyzed because of insufficient FNA sampling amount (n = 29), failed freezing storage of the FNA sample (n = 1), sample loss (n = 1), and insufficient water suppression or shimming (n = 3).

**Figure 2:**
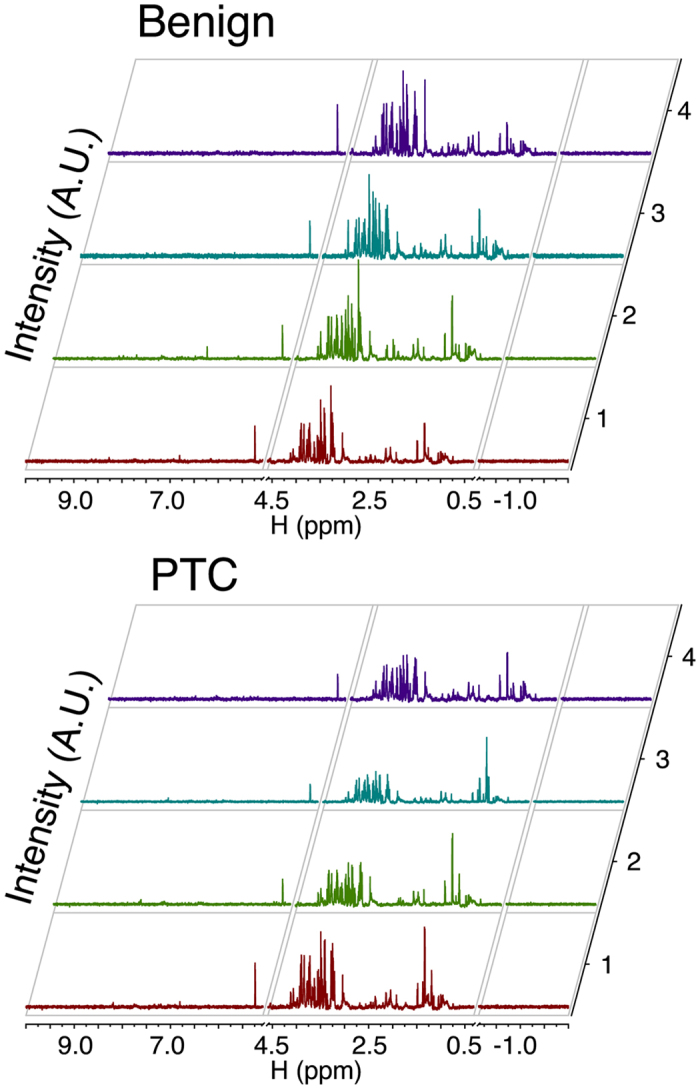
Representative metabolomic spectra from the fine needle aspiration specimens of benign and malignant (papillary thyroid carcinoma [PTC]) thyroid nodules.

Finally, percutaneous FNA samples of 104 nodules from 100 patients (M:F = 23:77; mean age, 52.9 ± 10.8 years; age range, 21–77 years; four patients with two nodules; 35 malignant nodules from 34 patients and 69 benign nodules from 66 patients) were analyzed using NMR (Fig. 2). The size of the thyroid nodules on US ranged from 3.5 to 27.6 mm (mean, 10.5 ± 6.2 mm) for malignant nodules and from 4.4 to 40.1 mm (mean, 15.2 ± 11.2 mm) for benign nodules. The information of 100 patients enrolled in this study was summarized in Table 1.

Of 35 malignant nodules, 25 nodules of 24 patients underwent surgery. All of these were confirmed to be malignant (papillary thyroid carcinoma).

Of 25 surgically confirmed papillary thyroid carcinomas, the results of BRAF^**V600E**^ mutation were available in 22 cancers. There were 18 BRAF^**V600E**^mutation-positive nodules and four mutation-negative nodules.

Only one of 69 benign nodules underwent surgery because of concomitant thyroid cancer. This case was diagnosed as nodular hyperplasia.

### NMR analysis of benign and malignant thyroid nodules

**Figure 3:**
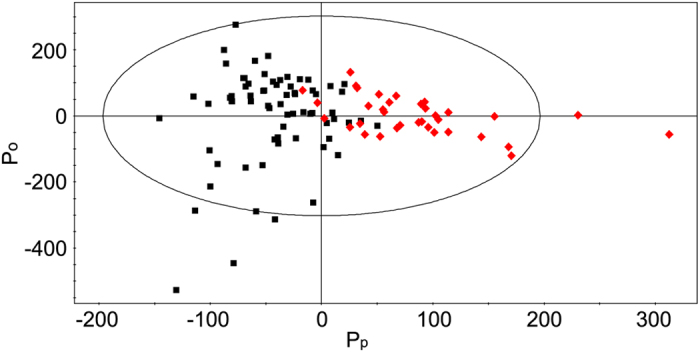
Orthogonal projections to latent structure-discriminant analysis (OPLS-DA) score plot showing the discrimination between benign and malignant (papillary thyroid carcinoma) thyroid nodules. The model was obtained using one predictive and one orthogonal components. Benign group: class 1 (black boxes), malignant group: class 2 (red diamonds) Pp represents the predictive component and Po represents the orthogonal component.

We performed multivariate statistical analysis to discriminate benign and malignant nodules. The orthogonal projections to latent structures-discriminant analysis (OPLS-DA) has been applied because it can discriminate groups in the presence of high structured noise or confounding factors. As indicated in Fig. 3, the OPLS-DA score plot (created using one predictive component and one orthogonal components) shows statistically significant discrimination between benign and malignant nodules with a Q^2^ value of 0.33 (R^2^Y = 0.59). In the prediction validation study with three-fold cross-validation, the accuracy, sensitivity, and specificity for diagnosing papillary thyroid carcinoma were 88.6%, 75%, and 95.6%, respectively.

### Metabolic differences between benign and malignant thyroid nodules

**Figure 4:**
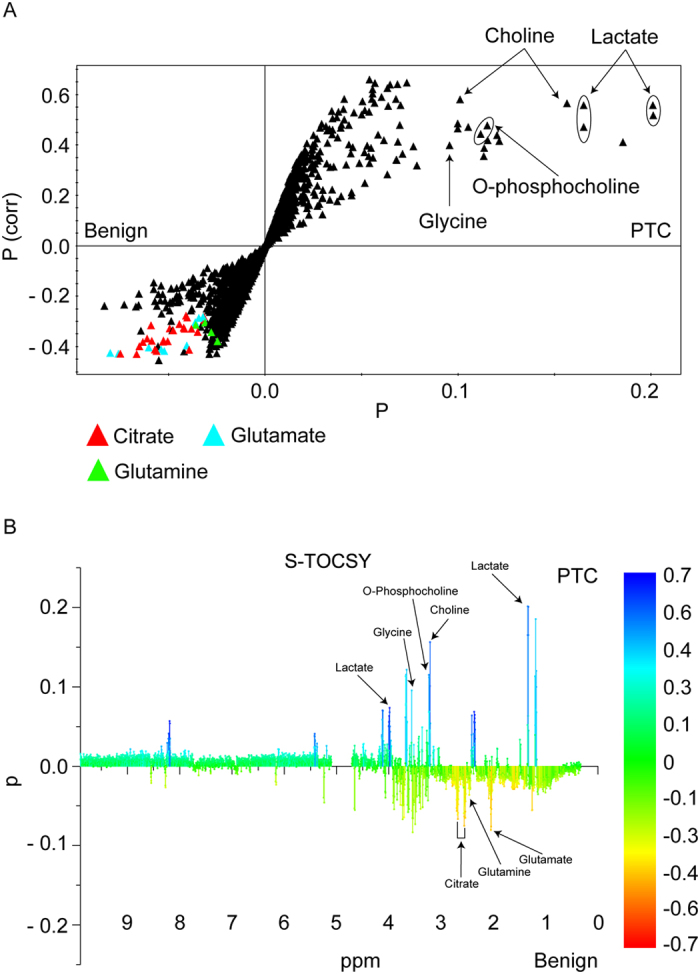
Identification of metabolites contributing to discriminating model. (**A**) OPLS-DA loading plot showing the metabolites of benign nodules and malignant nodules (papillary thyroid carcinoma) for marker identification. (**B**) OPLS loading plot (S-TOCSY) showing the model coefficients for each NMR variable. The signals are color coded according to their weights as a discriminator between benign and malignant thyroid nodules. Metabolites that significantly discriminate the two groups were annotated on the model coefficient plot.

According to OPLS-DA loadings of the predictive latent variable, the relative amounts of lactate (1.3 ppm), choline (3.2 ppm), O-phosphocholine (3.2 ppm), and glycine (3.6 ppm) were greater in malignant nodules than in benign nodules. The relative amounts of citrate (2.6 ppm), glutamine (2.1 ppm), and glutamate (2.0 ppm) were greater in benign nodules than in malignant nodules (Fig. 4). These findings corresponded with the significant metabolites identified in the unpaired Student *t* test (Table 2).

### Performance of metabolomic analysis in discriminating between benign and malignant thyroid nodules

**Figure 5:**
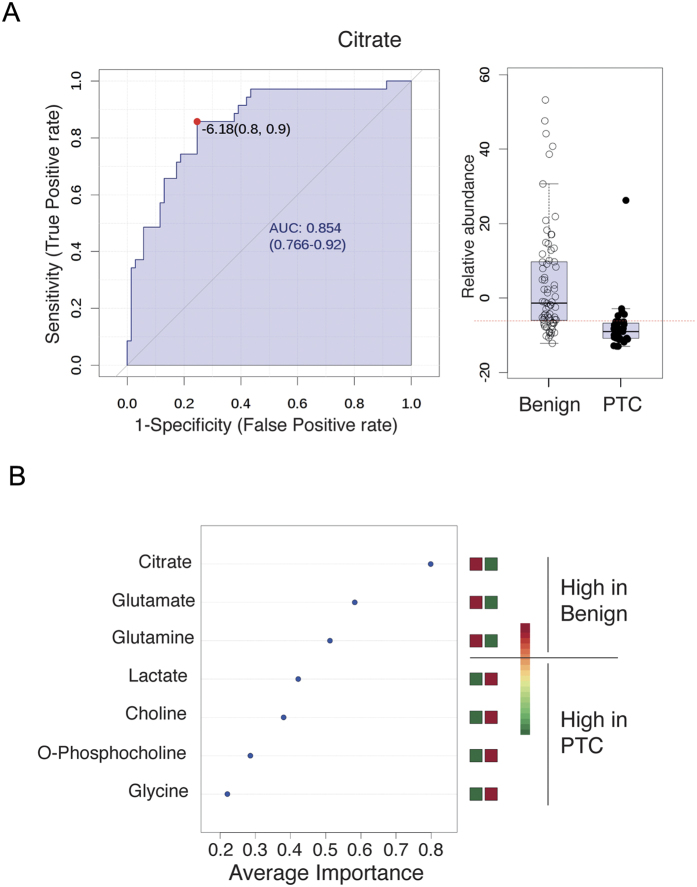
ROC analysis of citrate and multiple marker metabolites. (**A**) The ROC curve of citrate showing the ability as a discriminator of a thyroid nodule. (**B**) Multiple ROC curve analysis showing that all the seven metabolites had additive values in discriminating benign thyroid nodules from papillary thyroid carcinomas (PTC). The single most important discriminator was citrate which was more abundant in benign thyroid nodules than in PTC.

For evaluating the discriminating model, we performed the receiver operating characteristic (ROC) curve analysis. The grades predicted by the PLS-DA model showed that area under the curves (AUCs) ranged from 0.64 (glycine; not shown) to 0.85 (citrate). Citrate exhibited the sensitivity of 90% and specificity of 80% (Fig. 5A). To evaluate other marker metabolites, we performed multiple ROC analysis with seven metabolites and the result showed that citrate was the best discriminator (Fig. 5B).

### NMR analysis of BRAF ^V600E^ mutation-positive and negative papillary thyroid carcinomas

In the analysis of difference in metabolomic profiles between BRAF^**V600E**^mutation-positive papillary thyroid carcinomas and mutation-negative papillary thyroid carcinomas, there were no significant differences because two groups did not separated with negative Q^2^ value (−0.536) at OPLS-DA analysis.

## Discussion

To our knowledge, the current study enrolled the largest number of thyroid nodules for NMR analysis. It was also the first study showing that NMR analysis of percutaneous FNA specimens of thyroid nodules could be applied for preoperative diagnosis of thyroid cancer. Although some previous studies reported that preoperative *in vivo*^1^H MRS differentiated between benign and malignant thyroid nodules, this technique cannot be easily applied to patients with thyroid nodules. This is because thyroid nodules are not usually large enough to place voxel for *in vivo*^1^H MRS and may be influenced by susceptibility artifact due to their anatomical adjacency to the airway^21^,^23^.

Although metabolic analysis usually uses biofluids such as urine, blood, cerebrospinal fluid, and bile juice^15^,^17^,^19^,^20^, this study was the first to show that FNA specimens of thyroid nodules can be analyzed by NMR for preoperative diagnosis of malignancy. We also proved that only a small amount of FNA specimen sample (20–40 μL) is required for metabolic analysis. A few papers have reported that the metabolic spectral analysis of tissue and FNA specimens from surgically resected thyroid tissues did not significantly differ for diagnosis of papillary thyroid carcinoma^22^,^24^.

Although some specimens could not be used for metabolomic analysis in the present study, NMR analysis was performed in approximately 75% of all cases especially with only the remainder of the samples after allotment for conventional cytopathologic analysis. This indicates that the NMR approach is not an extra burden for patients in providing a better diagnosis.

The present study had lower statistical power than previous studies using postsurgical specimens, which had Q^2^ (R^2^Y) ranging from 0.37 (0.75) to 0.91 (0.82)^25^,^27^. This might be due to the small sample volumes and subsequent lower sensitivity of detection for NMR analysis of percutaneous FNA specimens. Bleeding and contamination with skin, subcutaneous fat, muscle, thyroid capsule, and normal thyroid parenchyma in the course of needle passage may have affected the NMR spectra.

Higher relative concentrations of lactate and choline, and lower relative concentrations of citrate, glutamine, and glutamate in malignant thyroid nodules were found in the present study using preoperative percutaneous FNA specimens. These results are generally concordant with those of several previous *in vivo*^1^H MRS studies and studies using surgical specimens^21^,^23^,^25^,^26^,^27^,^28^. This concordance suggests that preoperative metabolomic analysis could be a reliable test for characterizing the pre-operative metabolomic profiles of thyroid nodules.

Lactate concentrations are frequently increased in thyroid cancer and other malignant tumors^21^,^22^,^23^,^25^,^26^,^29^,^30^,^31^. Increased lactate level implicates an increase in the glycolytic pathway due to hypoxia or ischemia in tumor tissues or as a result of the Warburg effect^25^,^26^,^27^,^29^,^30^,^31^,^32^. Lactate was also proven to be a key factor in terms of cancer cell mobility^33^. Accelerated cancer cell metabolism has also been shown to produce more waste products, such as lactate, for extrusion and neutralization^25^,^32^.

Increased glycine in thyroid cancer may also result from increased glycolysis. Many recent studies reported that glycolytic intermediate metabolism plays an important role in tumorigenesis, and one of the main pathways is single carbon metabolism. Recently mitochondrial synthesis and consumption of glycine was proposed to be necessary for rapidly growing cancer cells. And glycine dehydrogenase (GLDC), which cleavages glycine and mediates folate cycle charging, are highly expressed on tumor promoting cells and its enhanced activity is associated with tumorigenesis. Therefore glycine could be one of important metabolites at tumorigenesis with its *de novo* synthesis and catabolism^33^,^34^,^35^.

Unlike the results of previous studies using postoperative surgical specimens, this study presented that choline was increased in thyroid cancer^25^,^27^. Because choline usually forms the phospholipids of cell membranes, malignant tissue cells that have increased multiplication and proliferation can also exhibit increased choline contents (choline phospholipid metabolism in tumor cells), which has been supported in many previous studies of thyroid cancer using MRS^21^,^22^,^23^,^26^.

Citrate was the most powerful discriminator in the present study. Since proliferating cells exhibit aerobic glycolysis and convert glucose to lactate (lactate fermentation, or the Warburg effect) at high levels, pyruvate-derived citrate synthesis in mitochondria may be reduced in these cells^26^,^36^,^37^. Several recent studies have reported that ATP citrate lyase, which uses citrate to synthesize acetyl CoA in a lipogenesis pathway for cell proliferation, is upregulated in some human cancers such as lung, colorectal, and ovarian cancers. In addition, these studies report that inhibition of ATP citrate lyase suppresses the proliferation of certain types of tumor cells^38^,^39^. As noted in these various cancers, proliferating thyroid cancer cells may also use citrate for lipogenesis and may have a lower concentration of citrate compared to that in benign cells.

Recent publications on cell metabolism emphasize that proliferating cells exposed to hypoxic conditions rely almost exclusively on reductive carboxylation of glutamine-glutamate-derived α-ketoglutarate for *de novo* lipogenesis^36^,^37^. Furthermore, some renal cell lines utilize this type of reductive glutamine metabolism even in the normoxic state^37^. Therefore, several research groups are currently studying the possibility of developing a glutaminase inhibitor as an anti-cancer drug. These observations from earlier studies could possibly explain why proliferating cells or cancer cells might have lower glutamine or glutamate levels than normal cells.

This study has several limitations. First, since the reference used for comparison was based on Bethesda categories II and VI, which show typical benign and malignant cytologic features, the real metabolic spectra of benign and malignant thyroid nodules might be different from our results. Further study with a larger number of nodules should be performed to validate the diagnostic reference.

Second, about 25% of samples (34/138) in the present study could not be used for metabolic analysis. The most important reason was insufficient remaining sample volume after allotment for conventional cytologic analysis. A more delicate preparation method is needed to ensure a sufficient sample amount for both conventional cytologic analysis and NMR analysis. However, in this study, as approximately one third of the thyroid nodules (n = 50) were less than 1 cm which are not usually indicated for FNA, this could also make sample volumes insufficient.

Third, all malignant samples were papillary thyroid carcinomas. Further studies are necessary to evaluate the metabolic profiles of different thyroid neoplasms, such as follicular adenoma, follicular thyroid carcinoma, medullary thyroid carcinoma, and anaplastic thyroid carcinoma.

Fourth, this study failed to reveal the correlation between metabolic spectra and BRAF^**V600E**^ mutation, a useful prognostic marker for papillary thyroid carcinoma. Further studies using a large number of thyroid nodules should be planned to correlate between various molecular markers and metabolic profiles.

Lastly, studies with a large number of patients should be undertaken to verify the usefulness of NMR analysis in various clinical applications including the nodules with insufficient or indeterminate results and how to use NMR analysis in conjunction with the current cytopapthologic analysis.

In summary, NMR analysis of preoperative percutaneous FNA specimens of thyroid nodules presented different metabolomic profiles for benign and malignant thyroid nodules in this study. NMR analysis of FNA specimens of thyroid nodules may be useful in the accurate and rapid preoperative diagnosis of thyroid cancer.

## Methods

### Ethics statement

This prospective study was approved by the institutional review board of Seoul National University Hospital. All the methods used in this study were carried out in accordance with the approved guidelines. Informed consent was obtained from all patients.

### Study population and acquisition of nuclear magnetic resonance data

From November 2012 to June 2013, 230 nodules of 214 consecutive patients underwent FNA for diagnosis of thyroid nodules and subsequent sampling for NMR analysis by 9 board-certified radiologists specializing in head and neck imaging (mean: 6.4 years, 3–16 years of experience). Patients who had a history of previous FNA, thyroid surgery, or thyroid hormonal treatment were excluded from the study. For patients with multiple thyroid nodules, only the nodules with different ultrasonographic features from one another and located in different thyroid lobes were included.

Under the guidance of high-resolution US machines (IU22, Philips Medical Systems, Bothel, WA; AixPlorer, Supersonic Imagine, Aix en Provence, France; Logiq9, GE Medical Systems, Milwaukee, WI) with 10–12MHz linear transducers, FNA was performed with up to 4 needle passes using capillary or aspiration FNA techniques according to the characteristics of thyroid nodules. Immediately after FNA procedures, aspiration specimens were smeared on a slide, fixed with alcohol, and stained with Papanicolaou for conventional cytologic analysis. The remainders of aspiration specimens were collected in Eppendorf tubes (20–40 μL). The tubes were kept in a dry-ice box and stored in a liquid nitrogen tank until metabolomic analysis was performed.

Cytopathologic analyses were done according to the Bethesda system^5^. Among the 6 categories, benign (category II) and malignant (category VI) thyroid nodules were chosen for NMR analysis. Furthermore, for surgically confirmed papillary thyroid carcinomas, the BRAF^**V600E**^ mutation status was analyzed by direct DNA sequencing and metabolomic profiles were analyzed between mutation positive and negative groups.

Samples from thyroid nodules were slowly thawed in an icebox, after which they were centrifuged at 13,000 rpm. The supernatant was collected with a pipette and placed in 1.7-mm SJ tubes with 0.25% trimethylsilane propionic acid (TSP) buffer in D_2_O to a final volume of 35 μL. The one-dimensional spectra of thyroid samples were measured using an NMR spectrometer (BrukerBiospin, AVIII700, Billerica, MA, USA) equipped with a 1.7-mm PATXI probe operating at a proton NMR frequency of 700.193 MHz.

The acquisition parameters were: pulse, CPMG; time domain size, 32,768; relaxation delay, 2 s; number of scans, 128; spectral width, 14,097 Hz; mixing time, 76 ms; and temperature, 25 °C. The lactate signal (**δ** = 1.342 ppm) was used as a reference value.

### Data processing

All time-domain NMR data underwent Fourier transformation, phase correction, and manual baseline correction. The resulting frequency-domain data were binned at 0.0031-ppm intervals to reduce the complexity of NMR data for pattern recognition. The signals were normalized (area normalization) against total integration values and TSP buffer to exclude the effects of different volumes and NMR measurement variations. Data was then converted into an ASCII text file. The regions corresponding to water (4.71–5.1 ppm) were removed from all spectra. Binning, normalization, and conversion were performed using an in-house Perl program.

### Statistical analysis

The signals in specific bins that showed significant differences (*P* value < 0.05) between benign and malignant groups in terms of area normalization values were determined using the unpaired Student *t* test. Thereafter, metabolites were identified using Chenomx (Spectral Database, Edmonton, Alberta, Canada) by fitting the experimental spectra (significant signals) to those in the database.

The resultant spectral data sets were then imported into SIMCA-P version 11.0 program (Umetrics, Umeå, Sweden), and mean centering was performed with Pareto scaling for multivariate statistical analysis. Furthermore, OPLS-DA was performed with one predictive component and two orthogonal components for benign and malignant thyroid nodules and also for BRAF^**V600E**^ mutation positive and negative papillary thyroid carcinomas.

Class discrimination models were created while ensuring that the cross-validated predictability value did not significantly increase to avoid over-fitting of the statistical model. Diagnostic performance was obtained by predicting three-fold cross-validation samples on the basis of a distinction model constructed using the rest of the samples. An *a priori* cut-off value of 0.5 was used to evaluate the prediction results^40^. An in-house written R script was used to identify the signals specific for each group by performing the Wilcoxon rank-sum test on all ppm variables (Q^2^ and R^2^). Eventually, the specific signals were compared to metabolites identified using the Chenomx data base.

The performance of the prediction derived from OPLS-DA modeling of NMR spectra was evaluated by computing the AUC using an open source ROC curve analysis tool for metabolomics data (ROC Curve Explorer & Tester, www. roccet.ca).

## Additional Information

**How to cite this article**: Ryoo, I. *et al*. Metabolomic analysis of percutaneous fine-needle aspiration specimens of thyroid nodules: Potential application for the preoperative diagnosis of thyroid cancer. *Sci. Rep.* **6**, 30075; doi: 10.1038/srep30075 (2016).

## Figures and Tables

**Table 1:** Demographic and pathologic characteristics of the patients.

	Papillary thyroid carcinoma	Benign thyroid nodule
Number of patients	34	66
Age (years)	50.0 ± 11.7	54.4 ± 10.1
Male/Female	10/24	13/53
T stage		
T1a	3	
T1b	0	
T2	0	
T3	21	
T4	0	
N/A*	10	
N stage		
N0	12	
N1a	11	
N1b	1	
N/A*	10	
BRAF^**V600E**^ mutation		
positive	18†	
negative	4	
N/A*	13	

Note - Unless otherwise specified, the data are the means ± standard deviations.

^*^N/A, Not available.

^†^18, one patient had two cancers with BRAF^**V600E**^ mutation and remaining 16 patients had one cancer per person.

**Table 2:** Relative concentration of metabolites in benign and malignant nodules.

Metabolite	Area normalization value		*P* value†
	Benign	PTC*	
Citrate	2.45 ± 1.37	1.33 ± 0.49	0.004
Glutamate	6.45 ± 1.58	5.50 ± 0.81	0.003
Glutamine	1.82 ± 0.52	1.59 ± 0.43	0.01
Lactate	6.89 ± 2.65	9.54 ± 3.15	0.003
Choline	1.94 ± 0.52	2.67 ± 0.92	0.0008
O-phosphocholine	2.72 ± 0.96	3.50 ± 1.12	0.002
Glycine	0.63 ± 0.31	0.83 ± 0.41	0.005

Note - Unless otherwise specified, the data are the means ± standard deviations.

^*^PTC, papillary thyroid carcinoma.

^†^*P* value for the comparison of means was calculated using the unpaired Student *t* test.
